# Type 2 Diabetes Mellitus in a Young Female Later Diagnosed as​​​​ Pancreatogenic Diabetes: The Dilemma in Classifying Diabetes Mellitus

**DOI:** 10.7759/cureus.50828

**Published:** 2023-12-20

**Authors:** Mahak Lamba, Ambuj Yadav, Deepak Bhagchandani, Himanshu Reddy, Vikas Chandra Vidyarthi

**Affiliations:** 1 Internal Medicine, King George's Medical University, Lucknow, IND; 2 Internal Medicine, Gastroenterology and Hepatology, King George's Medical University, Lucknow, IND; 3 Medicine, King George's Medical University, Lucknow, IND

**Keywords:** diagnostic dilemma, type 3 diabetes, atrophy of the pancreas, pancreatogenic diabetes, diabetes mellitus

## Abstract

Diabetes mellitus type 3c (DM3c) is a diabetes caused by pancreatic pathology. It occurs due to the destruction of the endocrine islet cells. Diabetes diagnosed at the age of 20-30 years share a common dilemma in segregating between the type of diabetes the patient has, as its management varies depending on the type of diabetes the patient is harboring. However, insulin remains the treatment of choice in later decades as the pancreatic reserves of beta cells exhaust, although it takes decades to happen.

We report a case of a woman who was diagnosed with diabetes mellitus at the age of 26, was on oral hypoglycemic agents (OHA), and was shifted to insulin therapy as she became non-responsive to OHA in a short span of six years, which was alarming. The patient presented to us with the chief complaints of recurrent abdominal pain that aggravated on taking meals and was associated with multiple episodes of vomiting for two months. Blood gas analysis on admission had no evidence of metabolic acidosis, urine ketones were negative, and a random blood sugar test (RBS:202) excluded the possibility of diabetic ketoacidosis. Serum amylase and serum lipase were within normal limits. Contrast-enhanced computed tomography (CECT) of the abdomen was suggestive of the atrophic pancreas with the non-dilated main pancreatic duct. Magnetic resonance cholangiopancreatography (MRCP) was done to rule out the congenital anomalies of the pancreas responsible for chronic pancreatitis, which showed no structural abnormalities. During our clinical workup, we postulated that the diabetes she was diagnosed with at the age of 26 was DM3c, i.e., pancreatogenic diabetes. The rapid shift of patients from OHA to subcutaneous insulin in a short span must be alarming to the physician managing diabetes and needs extensive workup to look upon the etiology of the same.

## Introduction

Diabetes mellitus (DM) is a disease characterized by inappropriately increased blood glucose levels. DM can present as type 1 diabetes, type 2 diabetes, maturity-onset diabetes of the young (MODY), pancreatogenic diabetes, gestational diabetes, neonatal diabetes, and secondary causes due to endocrinopathies, steroid use, etc. Type 1 DM affects children and adolescents, while type 2 DM affects middle-aged and older adults. The pathogenesis, etiologies, and presentations vary significantly, similar to their treatments [1].

Diabetes mellitus type 3c (DM3c) is mainly caused by chronic pancreatitis, accounting for nearly 75% of cases [2-5]. It can also occur during pancreatic manipulation in surgery for pancreatic cancer [4]. Alpha and beta cells are often targeted in the pancreas causing decreased glucagon and insulin levels. Blood glucose level often varies in these patients as the destruction of glucagon-producing alpha cells causes hypoglycemia, whereas the destruction of insulin-producing beta cells causes hyperglycemia [3].

Factors such as pancreatic calcifications, longer duration of the disease, and smoking often increase the chances of the patient developing chronic pancreatitis. Vitamin and mineral deficiencies are commonly encountered due to derangement in metabolic profile attributed to pancreatic disease. Low glycogen stores make the management of DM3c a bit challenging for the treating physician [6].

## Case presentation

**Figure 1:**
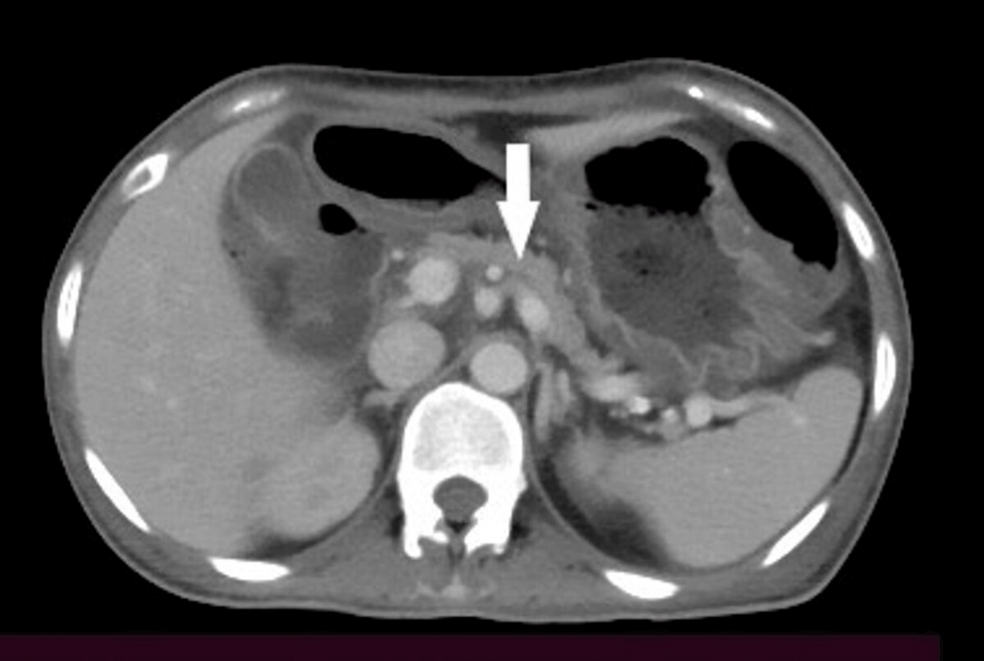
Arrow showing atrophied pancreas on CECT abdomen CECT: Contrast-enhanced computed tomography.

A 33-year-old female, who was a known diabetic for seven years on OHA and was referred to our hospital, presented to us with the chief complaints of recurrent abdominal pain that aggravated on taking meals and was associated with multiple episodes of vomiting for two months. The patient did not have any significant family history of diabetes and belonged to a low socioeconomic status. The patient had a history of intolerance to fatty meals and had bouts of diarrhea when consuming the same. The consistency of the stool has also changed over three years, which was often greasy, sticking to the stool pot, and difficult to flush associated with a foul smell. The patient took medications periodically for the latter under the care of a local practitioner. Vitals on presentation were blood pressure (BP): 120/70 mmHg and pulse rate (PR): 116/min. On physical examination, abdominal tenderness was present. Lab investigations suggested C-peptide: 0.26 ng/ml (<0.8 ng/ml), antinuclear antibody (ANA): negative, IgG4: 70.7, and decreased fecal elastase (<100 ug/g). Autoimmune antibodies related to type 1 diabetes were planned but could not be done due to affordability issues and the nonavailability of the same testing from our institution. CECT of the abdomen was suggestive of the atrophic pancreas with the non-dilated main pancreatic duct (Figure 1).

MRCP was done, which did not reveal any developmental malformation of the pancreas. The patient was earlier on OHA, which, according to the patient, maintained control for approximately three years. However, as the condition could no longer be controlled by OHA and the patient developed a nonhealing foot ulcer due to the uncontrollable state,​ the patient was shifted to insulin injections. Upon admission, the patient was given regular insulin and injection glargine, but the patient had multiple and recurrent episodes of daytime hypoglycemia when injection glargine was used; therefore, long-acting insulin was withdrawn, and the patient was then controlled and maintained on injection of regular insulin, which was attributed to the decreased levels of both glucagon and insulin levels due to the destruction of alpha and beta cells, respectively. During the hospital stay, the patient was managed conservatively by IV fluids and antibiotics. The patient improved clinically and was taken on premeal injection of regular insulin, along with pancreatic enzymes.

## Discussion

DM does not always warrant OHAs at diagnosis; pharmacotherapy revolves around its etiology. Type 2 DM is the most commonly prevailing diabetes in the community. The global prevalence of type 2 diabetes is estimated to increase to 7079 per 100,000 individuals by 2030 [7]. However, the answer to the non-visibility of something does not always echo with its mere absence; pancreatogenic diabetes is one such entity, which is often less thought and under-researched. The prevalence of pancreatogenic diabetes is 5%-10% among all diabetic patients in the Western population [3,5].

Suspicion of pancreatitis warrants extensive workup to look upon the etiology of the same. In some cases, the cause is elicited even on judicious history taking such as chronic alcohol consumption for many years, but sometimes, radiological parameters are advocated to elicit the cause, such as gallstone-induced pancreatitis. At times, the cause remains dormant and cannot be elicited, and is labeled as idiopathic pancreatitis. In a nationwide study conducted on patients between 1998 and 2007, idiopathic acute pancreatitis had an estimated 81,8025 admissions with a mean hospitalization of 5.6 days [8].

Intense control of hyperglycemia is advocated to achieve the goal of HbA1c < 7% for the better management of T3cDM. Better control of hyperglycemia would possibly aid in retarding the progression of the disease, preventing the risk of micro- and macrovascular complications [9]. Treatment modalities for pancreatogenic diabetes include both pharmacological and non-pharmacological treatment. Medical nutritional therapy (MNT) is helpful in the control of DM3c along with insulin therapy. MNT includes advice regarding having meals rich in soluble fiber, low in fat, and supplemented with oral enzyme replacement therapy. Pancreatic enzyme replacement helps to control the symptoms of steatorrhea, thus protecting against fat-soluble vitamin deficiency. Maintaining adequate levels of vitamin D is necessary to prevent the development of metabolic bone disease and osteoporosis [10]. The deficit of endogenous insulin from the beta cells is often compensated by exogenous insulin supplementation in the form of insulin therapy. Total pancreatectomy with islet autotransplantation (TPIAT) is considered the definitive treatment of recurrent acute or chronic pancreatitis for providing pain relief. Assessment of pancreatic endocrine reserve and functional β-cell mass should be done before TPIAT. Functional β-cell mass can be assessed biochemically by serum C-peptide levels [11].

## Conclusions

DM today is beyond the horizons of type 1 and type 2. Patients who initially respond to OHA may not always have type 2 DM. The rapid development of complications and progression of the disease must alarm the physician to conduct an extensive workup to look up the underlying etiology.
